# Regulation of arterial pressure by the paraventricular nucleus in conscious rats: interactions among glutamate, GABA, and nitric oxide

**DOI:** 10.3389/fphys.2012.00490

**Published:** 2013-01-09

**Authors:** Marli C. Martins-Pinge, Patrick J. Mueller, C. Michael Foley, Cheryl M. Heesch, Eileen M. Hasser

**Affiliations:** ^1^Department of Physiological Sciences, Center of Biological Sciences, State University of LondrinaLondrina, Brazil; ^2^Department of Biomedical Sciences, University of MissouriColumbia, MO, USA; ^3^Dalton Cardiovascular Research Center, University of MissouriColumbia, MO, USA; ^4^Department of Medical Pharmacology and Physiology, University of MissouriColumbia, MO, USA

**Keywords:** autonomic, sympathetic, NMDA, PVN, hypothalamus

## Abstract

The paraventricular nucleus (PVN) of the hypothalamus is an important site for autonomic and neuroendocrine regulation. Experiments in anesthetized animals and *in vitro* indicate an interaction among gamma-aminobutyric acid (GABA), nitric oxide (NO), and glutamate in the PVN. The cardiovascular role of the PVN and interactions of these neurotransmitters in conscious animals have not been evaluated fully. In chronically instrumented conscious rats, mean arterial pressure (MAP) and heart rate (HR) responses to microinjections (100 nl) in the region of the PVN were tested. Bilateral blockade of ionotropic excitatory amino acid (EAA) receptors (kynurenic acid, Kyn) in the PVN produced small but significant decreases in MAP and HR. GABA_A_ receptor blockade (bicuculline, Bic), and inhibition of NO synthase [(NOS), N-(G)-monomethyl-L-arginine, L-NMMA] each increased MAP and HR. The NO donor sodium nitroprusside (SNP) produced depressor responses that were attenuated by Bic. NOS inhibition potentiated both pressor responses to the selective EAA agonist, N-methyl-D-aspartic acid (NMDA), and depressor responses to Kyn. Increases in MAP and HR due to Bic were blunted by prior blockade of EAA receptors. Thus, pressor responses to GABA blockade require EAA receptors and GABA neurotransmission contributes to NO inhibition. Tonic excitatory effects of glutamate in the PVN are tonically attenuated by NO. These data demonstrate that, in the PVN of conscious rats, GABA, glutamate, and NO interact in a complex fashion to regulate arterial pressure and HR under normal conditions.

## Introduction

The paraventricular nucleus (PVN) of the hypothalamus is a critical site of integration for autonomic- and neuroendocrine-mediated cardiovascular responses (Swanson and Sawchenko, 1983). In addition to its role in the hypothalamoneurohypophysial system, the PVN is involved in regulation of the autonomic nervous system (Pyner and Coote, 2000; Patel et al., 2001; Li and Patel, 2003; Coote, 2004). Morphological and electrophysiological studies have shown that the PVN is reciprocally connected to regions of the brain that are involved in cardiovascular regulation (Saper et al., 1976; Swanson and Sawchenko, 1983). Importantly, the PVN projects to both the intermediolateral cell column of the spinal cord and the rostral ventrolateral medulla (RVLM), regions critical in control of the sympathetic nervous system (Badoer, 1996; Pyner and Coote, 2000; Hardy, 2001).

Nitric oxide (NO) has been shown to act as a gaseous neuromodulator to influence synaptic function in the central nervous system (Bredt et al., 1990; Hirooka et al., 1996; Martins-Pinge et al., 1997; Zanzinger, 1999). NO synthase (NOS) is expressed in the PVN (Zhang et al., 1998; Kantzides and Badoer, 2005; Mueller et al., 2006) and NO appears to play a role in endocrine and autonomic regulation of cardiovascular responses. *In vitro* studies indicate that neuronal activity within the PVN is modulated by NO (Li et al., 2003; Stern, 2004). In addition, administration of an NO donor into the PVN decreases renal sympathetic nerve discharge, arterial pressure, and heart rate (HR; Horn et al., 1994; Zhang and Patel, 1998). Conversely, microinjection of NOS blockers into the PVN produces pressor and sympathoexcitatory responses (Zhang and Patel, 1998; Wang et al., 2005). These data, primarily from anesthetized animals, suggest that NO has a tonic effect within the PVN to inhibit resting sympathetic activity and arterial pressure.

The effects of NO within the PVN appear to involve a complex interaction with the neurotransmitters glutamate and gamma-aminobutyric acid (GABA). It has been proposed that activation of the NMDA subtype of ionotropic glutamate receptors increases release of NO in the PVN, which then negatively modulates NMDA-mediated increases in sympathetic nerve discharge (Li et al., 2001). Depressor and sympathoinhibitory responses to NO donors are blocked by the GABA_A_ receptor antagonist bicuculline (Bic; Zhang and Patel, 1998). This is consistent with the concept that NO may blunt responses to exogenous NMDA by increasing GABA transmission. However, it is not known whether endogenous NO tonically modulates basal excitatory effects of glutamate within the PVN.

The role of NO is known to differ dramatically during different levels of neuronal activity and under a variety of physiological and pathophysiological conditions (Villar et al., 1994; DiCarlo et al., 2002; Felder et al., 2003; Li and Patel, 2003; Mueller et al., 2003, 2006; Heesch et al., 2009). However, the interactions among NO, GABA, and glutamate have been studied primarily in anesthetized animals (Zhang and Patel, 1998; Li et al., 2001; Patel et al., 2001; Akine et al., 2003). Anesthesia is well-known to alter neurotransmission, including both GABAergic and glutamatergic neurotransmission (Franks and Lieb, 1982; Jin et al., 2009; Olsen and Li, 2011), and autonomic and cardiovascular regulation (Schadt and Ludbrook, 1991; Moffitt et al., 1998, 1999; Araújo et al., 1999; Sakima et al., 2000; Machado, 2001). Furthermore, anesthesia alters cardiovascular responses mediated by the PVN (Kannan et al., 1987, 1989). Given this evidence, it is apparent that GABA, glutamate, and NO function may differ in the conscious state. Therefore, it is critical to evaluate the relative roles of GABA, glutamate, and NO, and their interactions, in conscious animals in order to determine the importance of these transmitters in the PVN in control of the cardiovascular system in the conscious state.

This study in conscious rats tested the hypothesis that, in regard to control of arterial pressure, both NO and GABA in the PVN are tonically inhibitory, while glutamate is tonically excitatory. We also hypothesized that the tonic inhibitory effects of NO require GABAergic mechanisms within the PVN. Finally, we tested the hypothesis that endogenous NO modulates the tonic excitatory effects of glutamate.

## Experimental procedures

All procedures were performed according to the guidelines stated in the National Institutes of Health “Guide for the Care and Use of Laboratory Animals.” All protocols were approved by the University of Missouri-Columbia Animal Care and Use Committee. Twenty-nine male Sprague-Dawley rats (280–350 g, Harlan Sprague Dawley, Indianapolis, IN) were used in these studies. The rats were allowed to adapt in house for at least 1 week before any instrumentation. Rats were allowed access to water and food (Formulab Diet, 0.28% sodium, Purina, St. Louis, MO) *ad libitum*.

### Instrumentation

Initially, rats were anesthetized with Isoflurane [AErane, Baxter, Deerfield, IL (5% in 100% O_2_, 2 L per minute for induction and maintenance at 2–2.5%)], placed in a stereotaxic apparatus (Kopf, Tujunga, CA) and using aseptic technique bilateral guide cannulae (22 gauge) directed to the region of the PVN were implanted. Coordinates for cannulae placement were similar to previous work (Kenney et al., 2001; Li et al., 2001; Zhang et al., 2002; Chen and Toney, 2003): 1.8 mm posterior to bregma, 0.5 mm lateral to midline, and 7.6 mm below the surface, with the incisor bar set to 5 mm above the interaural plane and the cannula vertical. Studies from our laboratories have shown similar coordinates to produce injections sites localized to the PVN (Kvochina et al., 2009; King et al., 2012). Rats were allowed to recover for 12 days, anesthetized again with Isoflurane and aseptically instrumented with arterial and venous catheters for measurement of arterial pressure and HR and injection of drugs, respectively. The catheters were routed subcutaneously to the nape of the neck, exteriorized, and secured to the skin with suture; catheters were filled with heparinized saline (100 U/ml) and sealed with a stylet. Rats were allowed to recover for 2 days before the beginning of experiments.

### Experimental protocols

On the day of experimentation, arterial pressure, mean arterial pressure (MAP), and HR were monitored (PowerLab; ADInstruments, Colorado Springs, CO) for at least 30 min to ensure stable baseline parameters prior to any experimental manipulation. Microinjections were made using stainless steel injectors (33 gauge) connected by PE tubing to a 0.5 μl syringe (Hamilton, Reno, NV). Injectors were filled with the appropriate solution and inserted into the guide cannulae prior to injection. Protocols designed to evaluate tonic effects of endogenous transmitters utilized bilateral microinjections of appropriate agents to block the effects of the specific transmitter. Because the time course of the agonists used is relatively short, protocols examining effects of agonists utilized unilateral microinjections. As in other studies (Chen and Toney, 2003; Chen et al., 2003; Li and Pan, 2007a), the PVN initially was identified in all rats by pressor responses to Bic (1 mM) with mild or no behavioral response.

#### Tonic excitation of the PVN

Tonic effects on MAP and HR of ionotropic glutamate receptor activation in the PVN in conscious rats were evaluated by examining responses to bilateral microinjection of kynurenic acid [Kyn, 40 mM; 100 nl, (Li and Pan, 2007a)], a general ionotropic glutamate receptor antagonist. In control experiments, similar volumes of saline (0.9%) were injected bilaterally.

#### Tonic inhibition of the PVN

These protocols examined the tonic effects of NO and GABA_A_ receptor activation within the PVN on cardiovascular function. Bilateral microinjections (100 nl) of L-NMMA [N-(G)-monomethyl-L-arginine, 2 mM (Li et al., 2001), a NOS inhibitor, or Bic 1 mM, (Li et al., 2006)], a GABA_A_ receptor antagonist, were made in the PVN and hemodynamic responses monitored.

#### Transmitter interactions

To examine the interactions among NO, GABA, and glutamate in the PVN of conscious rats, the response to manipulation of the different receptor systems was evaluated in the presence and absence of other systems. Hemodynamic responses to unilateral microinjection of the NO donor sodium nitroprusside [SNP, 1 M; 100 nl, (Zhang and Patel, 1998) were evaluated alone and in the presence of Bic (1 mM; 100 nl), or saline (0.9%; 100 nl)]. Similarly, the effects of unilateral microinjections of the selective ionotropic glutamate receptor agonist N-methyl-D-aspartate [NMDA, 1 mM; 100 nl, (Li et al., 2001)] were evaluated alone and in the presence of the NOS inhibitor L-NMMA (2 mM; 100 nl).

To examine the interaction of tonic endogenous ionotropic glutamate receptor activation with endogenous NO, the response to bilateral PVN injection of Kyn (40 mM, 100 nl) was evaluated in the presence and absence of inhibition of NOS with L-NMMA (2 mM; 100 nl). Finally, the interaction of endogenous glutamate and GABA was evaluated. Hemodynamic effects of bilateral blockade of GABA_A_ receptors by microinjection of Bic (1 mM; 100 nl) were examined alone and in the presence of blockade of ionotropic glutamate receptors with Kyn (40 mM; 100 nl).

The time allowed for recovery following the initial administration of a specific agent was based on previous studies showing recovery, as well as repeatable responses (Li et al., 2001; Chen and Toney, 2003; Chen et al., 2003). The second administration of each drug was made 5 min after antagonist administration. A maximum of 3 drug administrations was performed in each PVN and drugs were applied using a microsyringe connected by PE tubing to an injector. At the end of the experiment, Pontamine sky blue dye (2%; 100 nl) was microinjected into the experimental site of the brain for subsequent histological estimation of injection sites.

**Figure 1:**
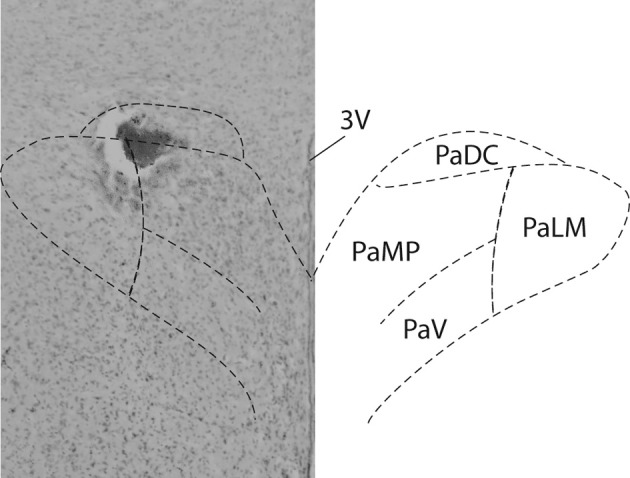
**PVN injection site.** Brightfield photomicrograph (Left) indicates the location of an injection site within the PVN of an individual rat. 100 nl of the dye Pontamine sky blue was injected at the conclusion of all experiments. A schematic of the PVN (Paxinos and Watson, 2007) is superimposed for reference.

At the end of the experiment, rats were euthanized by overdose of sodium pentobarbital; guide cannulae were detached from their cemented position on the skull and brains were fixed in 4% formaldehyde for at least 48 h. Serial coronal sections (40 μm) were cut using a cryostat, thaw mounted on microscope slides and stained with 1% aqueous neutral red. Figure 1 shows the injection site within the PVN in one rat. Unfortunately, in most rats removing the guide cannulae prior to fixation of the brains produced non-specific damage. However, injection tracts, the rostral-caudal spread of the dye and dye spots were confined to the brain parenchyma in regions consistent with PVN injection sites reported in previous studies from our laboratories and others (Martin and Haywood, 1998; Kenney et al., 2001; Li et al., 2001; Chen et al., 2003; Kvochina et al., 2009; King et al., 2012).

### Drugs

Bic methiodide, SNP, L-NMMA, and Kyn were obtained from Sigma Chemical (St. Louis, MO). NMDA was obtained from Tocris (St. Louis, MO). With the exception of Kyn, all drugs were dissolved in sterile saline. Kyn was first dissolved in a few drops of 1 N NaOH before being diluted in saline. All drugs were pH adjusted to 7.3–7.5 and filtered before microinjection. Doses of drugs for microinjection were taken from previous studies (Martin et al., 1991; Zhang and Patel, 1998; Chen and Toney, 2001; Chen et al., 2003; Li et al., 2006; Li and Pan, 2007a,b). Most drugs used for microinjection were relatively short-acting with recovery typically within 30–45 min or less.

### Analysis of data

All results are expressed as mean ± SEM. Responses to drugs are expressed as the difference between the basal value and the peak increase or decrease following each dose of drug. Student's two-tailed paired *t*-test was used for comparison of hemodynamic parameters between baseline conditions and after drug. We also used Student's two-tailed paired *t*-test to compare the response (change) to two different drugs or to a given drug in the presence and absence of another perturbation in the same rat. *P* < 0.05 was considered to indicate statistical significance.

## Results

### Baseline hemodynamic parameters

There were no significant differences in baseline hemodynamic parameters before any protocols involving microinjection of substances into the PVN in conscious rats (Table 1). Average baseline MAP was 117 ± 2 mmHg, and HR was 347 ± 4 bpm (*n* = 29). As described by others (Martin et al., 1991), after microinjection of Bic and NMDA into the PVN, we often observed mild behavioral responses, including alerting and facial grooming. These behaviors were not aggressive and were distinctly different from the defense responses described after activation of the posterior hypothalamus (Shekhar and DiMicco, 1987; Wible et al., 1988).

**Table 1:** **Mean arterial pressure (MAP) and heart rate (HR) before and after drugs microinjected in the PVN**.

**Groups**	**Before**		**After**	
	**MAP (mmHg)**	**HR (bpm)**	**MAP (mmHg)**	**HR (bpm)**
Bic (*n* = 7)	119 ± 6	346 ± 9	141 ± 5^*^	467 ± 17^*^
L-NMMA (*n* = 6)	113 ± 5	332 ± 10	133 ± 2^*^	410 ± 23^*^
Kyn (*n* = 4)	128 ± 6	371 ± 7	121 ± 4^*^	354 ± 8^*^
SNP (*n* = 6)	120 ± 2	344 ± 16	107 ± 3^*^	385 ± 11^*^
SNP (after Bic; *n* = 6)	121 ± 4	355 ± 17	114 ± 5^*^	395 ± 21^*^
Bic (after Kyn; *n* = 6)	125 ± 4	357 ± 14	134 ± 5^*^	408 ± 15^*^
Kyn (after L-NMMA; *n* = 4)	134 ± 8	383 ± 8	121 ± 7^*^	354 ± 14
NMDA (*n* = 4)	124 ± 2	361 ± 12	135 ± 2^*^	446 ± 17^*^
NMDA (after L-NMMA; *n* = 4)	124 ± 3	364 ± 5.1	142 ± 2^*^	466 ± 11^*^

* *p < 0.05 compared to values before microinjection*.

### Tonic ionotropic glutamatergic excitation within the PVN

**Figure 2:**
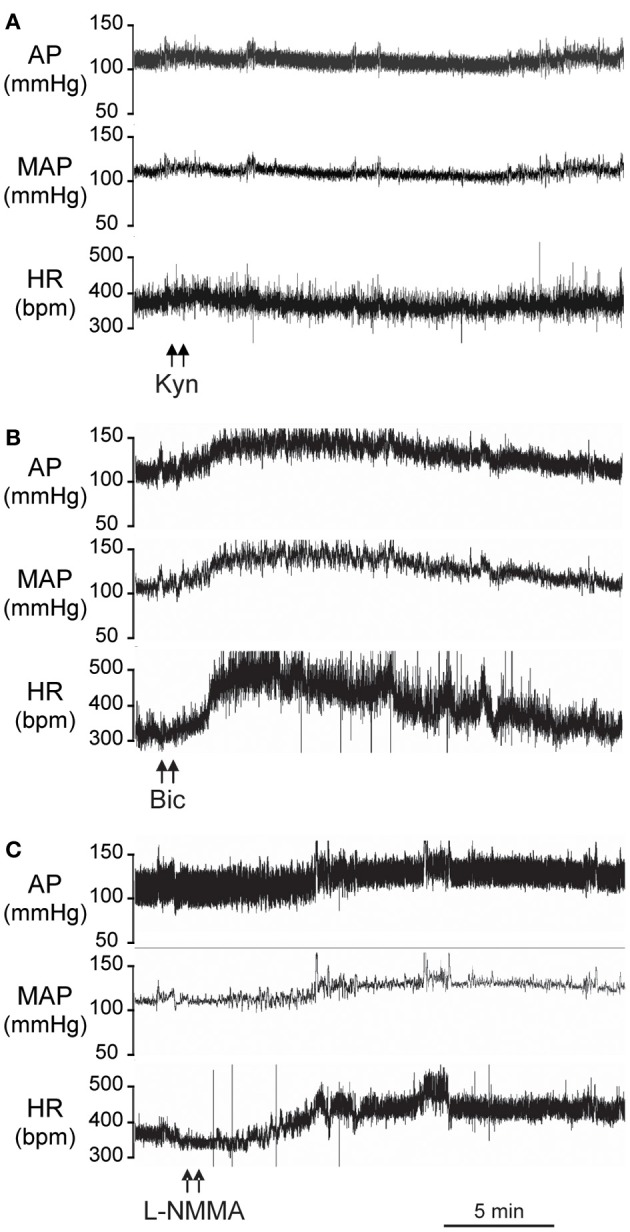
**Original records from individual conscious rats showing the arterial pressure (AP), Mean AP (MAP), and heart rate (HR) responses to bilateral microinjections (100 nl) into the PVN of: (A) Kynurenic acid (Kyn, 40 mM); (B) Bicuculline (Bic, 1 mM); (C) L-NMMA (2 mM)**.

Figure 2A is an original record showing MAP and HR responses to bilateral microinjection of the broad spectrum excitatory amino acid (EAA) receptor antagonist, Kyn, in the PVN of a conscious rat. Bilateral blockade of ionotropic glutamate receptors with Kyn (*n* = 4) produced a small but significant decrease in MAP (−6 ± 2 mmHg) and HR (−15 ± 3 bpm); Table 1. Bilateral microinjection of physiological saline (*n* = 5) produced no significant effect on MAP (−1 ± 1 mmHg) or HR (−4 ± 17 bpm).

### Tonic inhibition within the PVN

**Figure 3:**
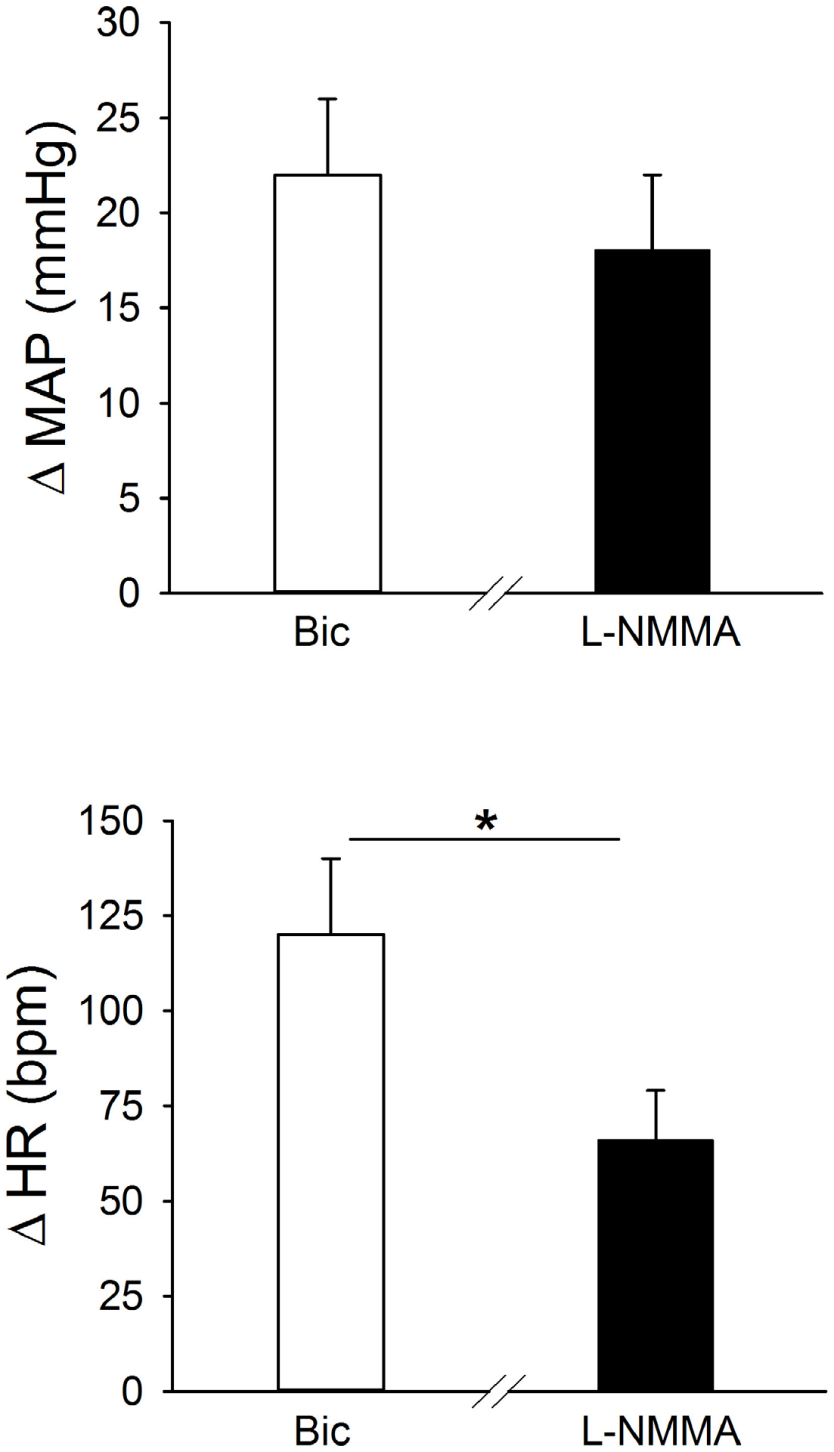
**Effects of bilateral blockade in the region of the PVN of GABA_A_ receptors (bicuculline; Bic, 1 mM; open bars; *n* = 7), or NO synthase (NOS) (L-NMMA; 2 mM, filled bars; *n* = 6) on MAP and HR in separate groups of conscious rats.** Bilateral microinjection of both Bic and L-NMMA significantly increased MAP and HR. The pressor response to Bic and L-NMMA was similar, while the tachycardic response to L-NMMA was significantly less than the response to Bic (^*^p ≤ 0.05).

Original tracings of hemodynamic responses to bilateral microinjection of the GABA_A_ receptor antagonist, Bic, or the NOS inhibitor, L-NMMA, into the PVN of individual conscious rats are shown in Figures 2B,C, respectively. Group data (Figure 3, Table 1) indicate that bilateral disinhibition of the PVN by microinjection of Bic (*n* = 7) produced a significant increase in both MAP and HR. Bilateral blockade of NOS in the PVN with L-NMMA (*n* = 6) also induced a pressor response accompanied by an increase in HR. The pressor responses to Bic and L-NMMA were not significantly different, but the HR effects were significantly greater for Bic.

### Activation of GABA_A_ receptors contributes to inhibitory effects of nitric oxide in the PVN of conscious rats

Initially, responses to two concentrations of the NO donor, SNP, were determined and repeatability of the response to multiple injections of SNP was tested. Unilateral microinjection of SNP (0.5 M and 1 M; *n* = 5) into the PVN produced rapid dose-related decreases in MAP (−11 ± 2 mmHg and −17 ± 3 mmHg, respectively). The depressor response was associated with tachycardia (51 ± 15 and 54 ± 3 bpm, respectively). Responses to unilateral microinjection of SNP (0.5 M) were repeatable and not altered after microinjection of saline into the PVN (MAP: −8 ± 2 vs. −10 ± 2 mmHg; HR: 45 ± 5 vs. 32 ± 8).

**Figure 4:**
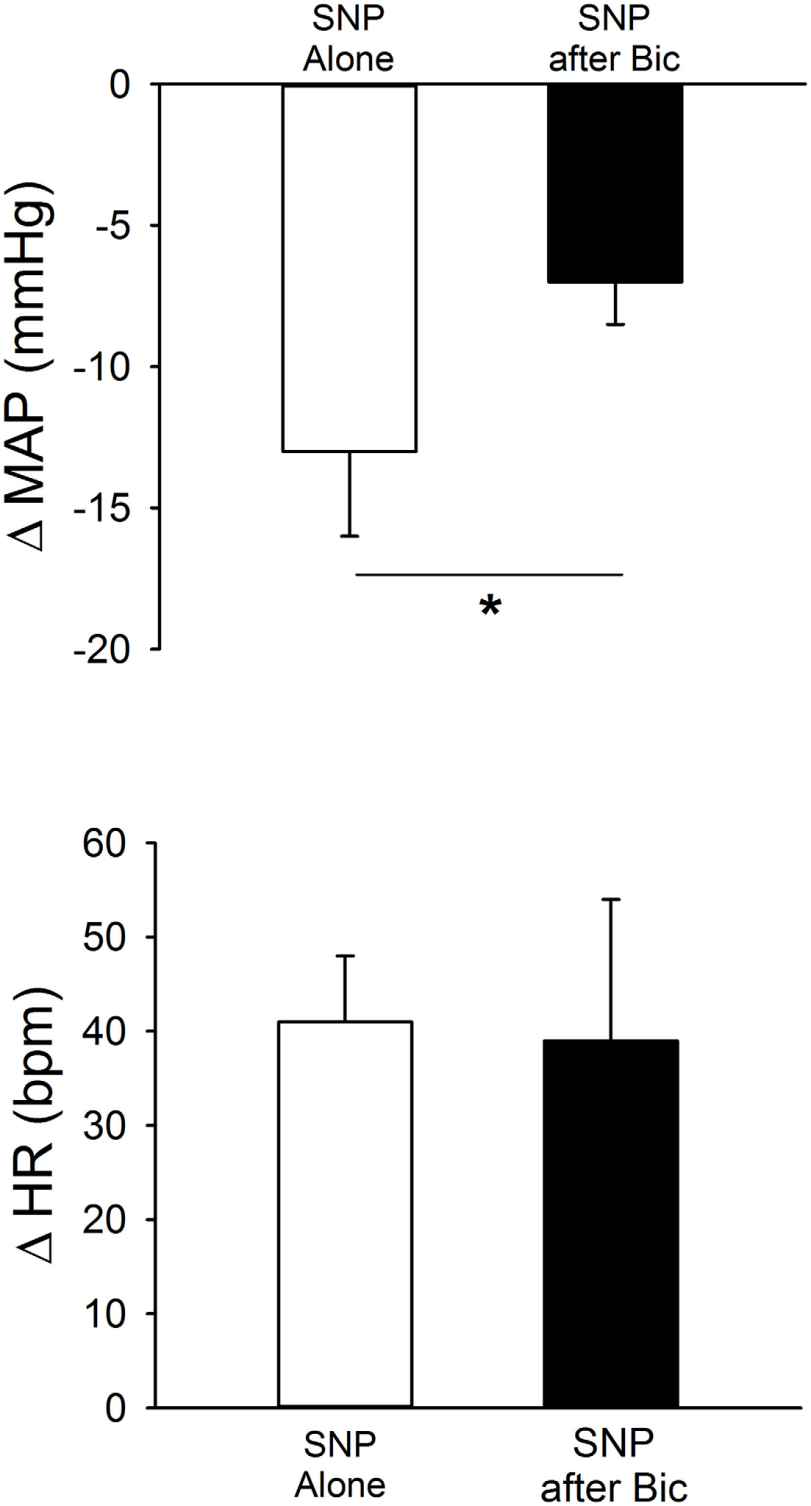
**MAP and HR effects of unilateral microinjection of the NO donor sodium nitroprusside (SNP, 1.0 M) into the PVN of conscious rats (*n* = 6).** SNP was injected before (open bars) and after (filled bars) blockade of GABA_A_ receptors with bicuculline (Bic, 1 mM). SNP alone produced significant decreases in MAP, accompanied by tachycardia. Bic significantly attenuated the depressor effect of SNP (^*^p ≤ 0.05) without altering the tachycardia.

In separate rats (*n* = 6) responses to SNP (1 M) were determined before and after injection of Bic (Figure 4, Table 1). Unilateral blockade of GABA_A_ receptors with Bic initially tended to produce small increases in MAP and HR; however, 5 min later hemodynamic parameters had returned to control. Importantly, baseline MAP and HR before injection of SNP alone or injection after unilateral Bic were not statistically different (Table 1). In the presence of Bic, the MAP response to SNP was attenuated by ~50% (Figure 4). The tachycardic response produced by SNP was not altered by previous Bic microinjection.

### Nitric oxide attenuates tonic glutamatergic excitation of the PVN

**Figure 5:**
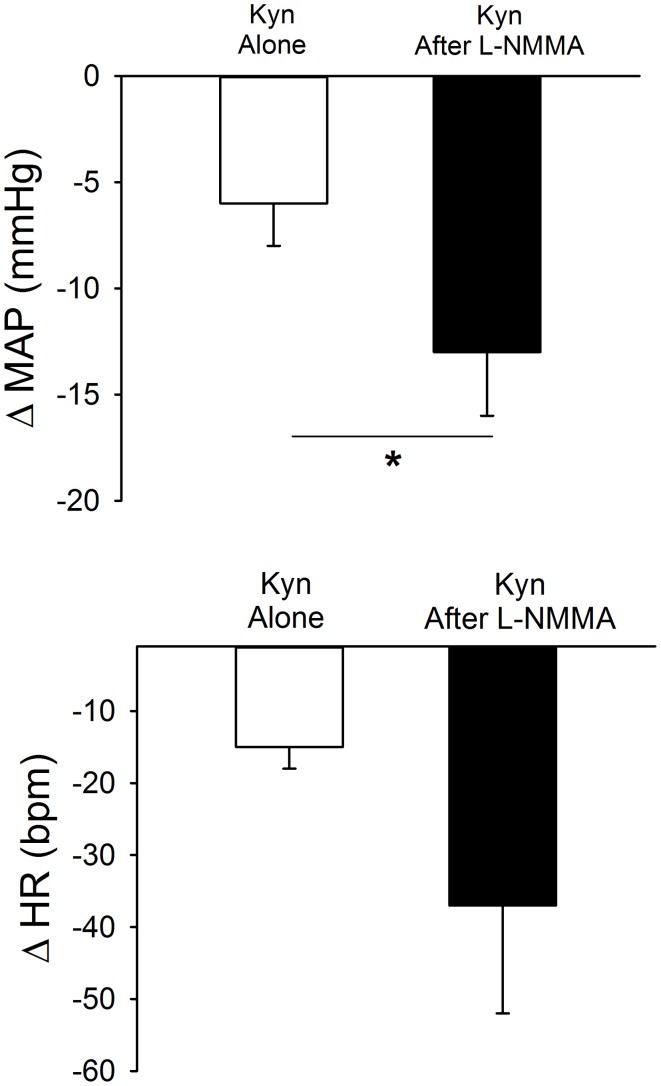
**Effects of bilateral PVN blockade of ionotropic glutamate receptors using kynurenic acid (Kyn, 40 mM) alone (open bars) or after bilateral inhibition of NOS with L-NMMA (2 mM, filled bars) in conscious rats (*n* = 4).** Bilateral microinjection of Kyn produced small but significant decreases in MAP and HR. Prior injection of L-NMMA significantly enhanced depressor responses to Kyn (^*^p ≤ 0.05) with no significant effect on HR responses.

As in the previous experiments, bilateral microinjection of Kyn into the PVN produced small but significant decreases in MAP and HR (Figure 5). After a 45 min recovery period, bilateral microinjection of Kyn was repeated in the presence of bilateral NOS blockade (L-NMMA). The baseline MAP and HR 5 min after L-NMMA, immediately before the second Kyn microinjection, were not different from the baseline before Kyn alone (Table 1). L-NMMA enhanced the depressor effect of ionotropic glutamate receptor blockade on MAP without significantly altering the HR responses (Figure 5).

**Figure 6:**
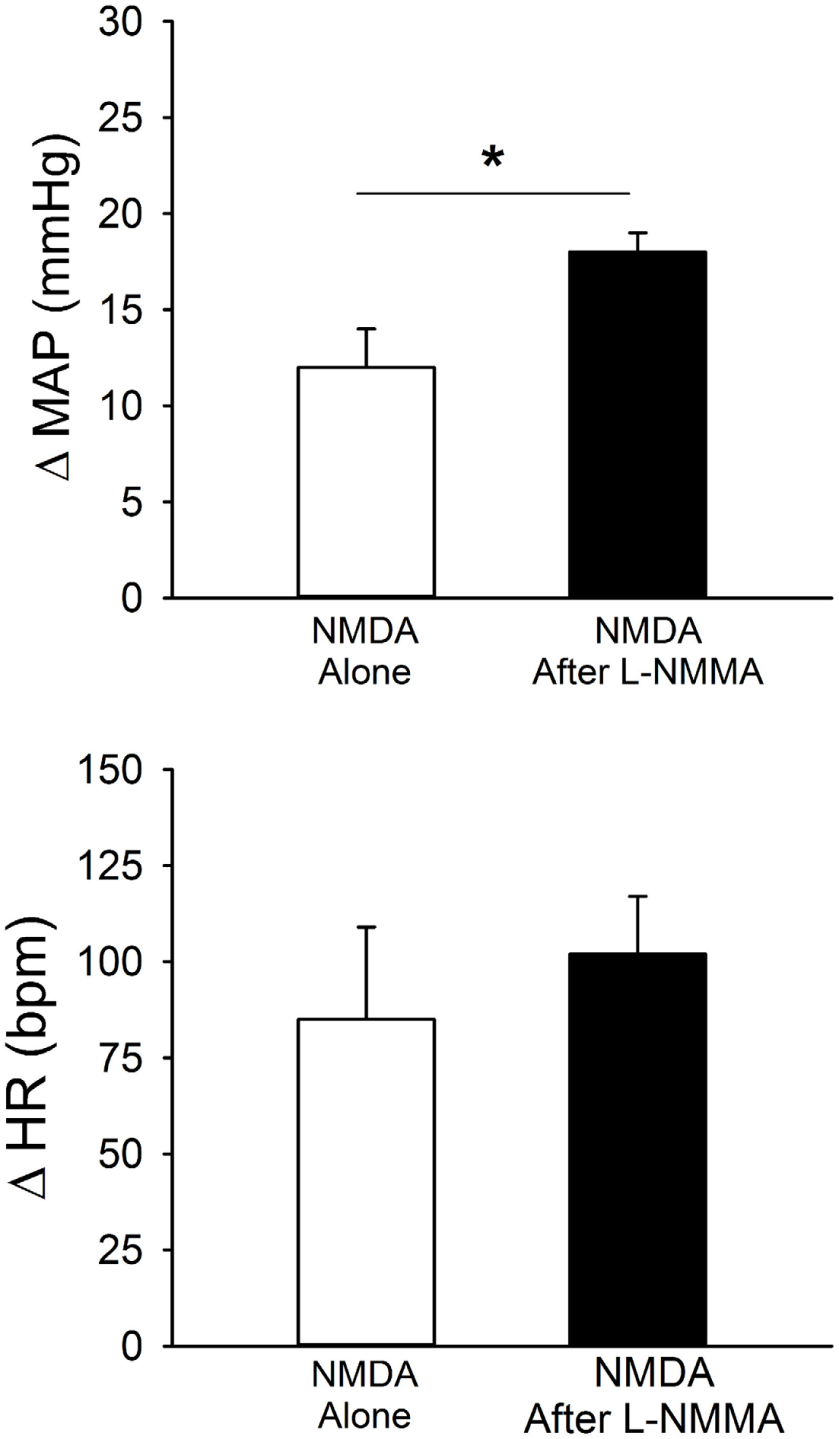
**MAP and HR effects of unilateral microinjection of the selective ionotropic glutamate receptor agonist N-methyl-D-aspartic acid (NMDA, 1 mM) into the PVN of conscious rats (*n* = 4).** NMDA was injected before (open bars) and after (filled bars) blockade of NOS with L-NMMA (2 mM). NMDA alone produced significant increases in MAP and HR. Pressor responses to NMDA were significantly enhanced by prior injection of L-NMMA (^*^p ≤ 0.05).

Unilateral microinjection of the ionotropic glutamate agonist, NMDA, into the PVN of conscious rats produced an increase in MAP and HR (Figure 6, Table 1). Unilateral microinjection of L-NMMA did not alter baseline cardiovascular parameters. However, during NOS inhibition the pressor response to NMDA was significantly augmented (Figure 6), consistent with a role of NO to blunt the excitatory effects of NMDA receptor activation. The tachycardia in response to NMDA was not significantly altered by L-NMMA.

### Tonic glutamatergic input contributes to excitation of the PVN due to GABA_A_ receptor blockade

**Figure 7:**
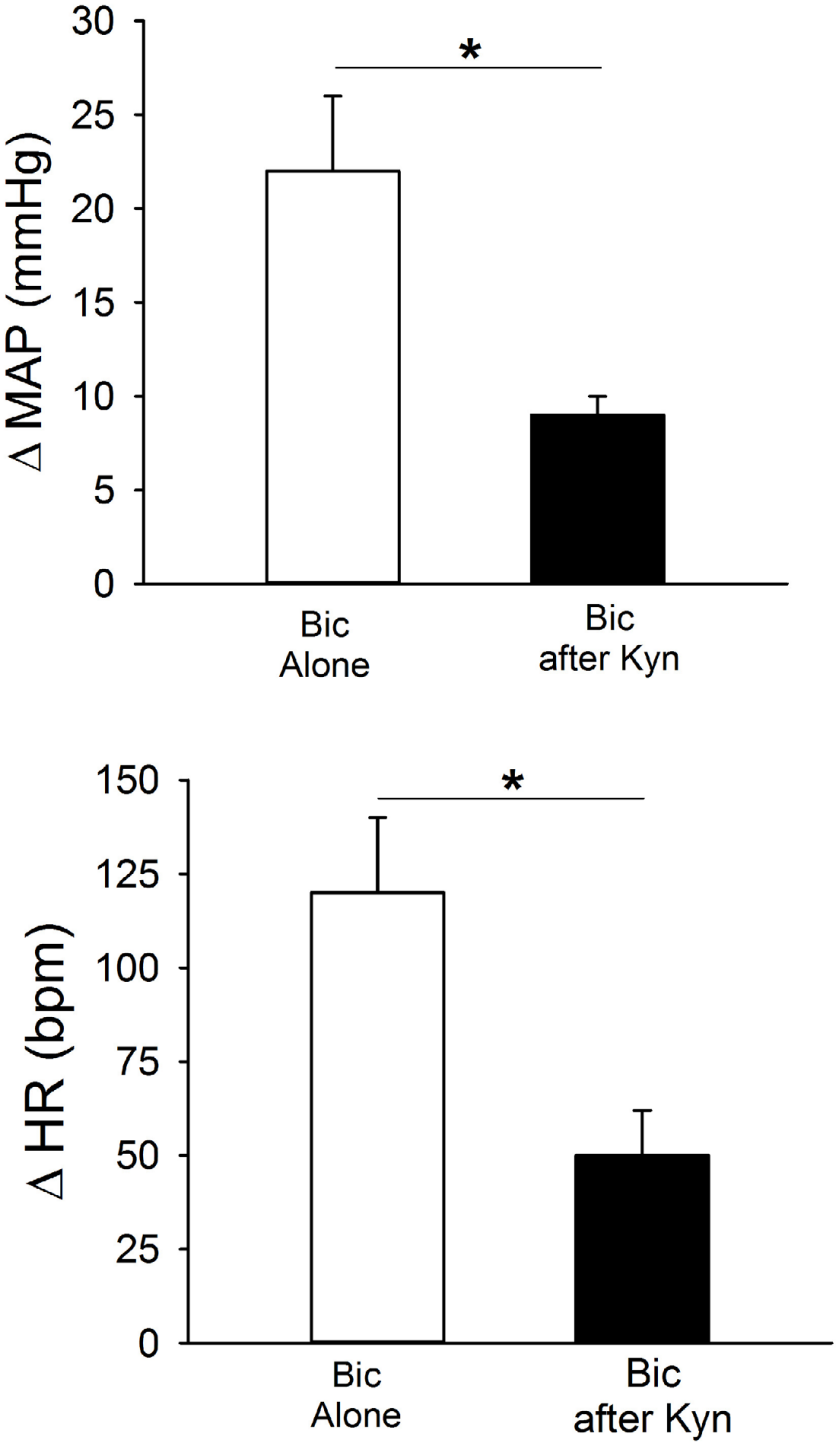
**Effects of bilateral blockade within the PVN of GABA_A_ receptors with bicuculline (Bic, 1 mM) on MAP and HR in conscious rats (*n* = 6) alone (open bars) or after blockade of ionotropic glutamate receptors with kynurenic acid (Kyn, 40 mM; filled bars).** Bilateral microinjection of Bic alone produced significant increases in MAP and HR. Pressor and tachycardic responses to Bic were significantly attenuated by prior microinjection of Kyn (^*^p ≤ 0.05).

Figure 7 contains mean data demonstrating responses to bilateral Bic before and after ionotropic glutamate receptor blockade. Bilateral PVN microinjection of Bic produced significant increases in MAP and HR. After recovery, Kyn was injected bilaterally, and 5 min later Bic was injected a second time. Following ionotropic glutamate receptor blockade, the pressor and tachycardic responses to Bic were significantly attenuated.

## Discussion

### Purpose

This study was designed to examine, in conscious rats, the influence of the PVN on arterial pressure and HR and to evaluate potential interactions of NO, glutamate, and GABA in mediating these effects. We also tested the hypothesis that endogenously released NO blunts the tonic excitatory effects of glutamate within the PVN. The use of conscious rats is an important aspect of these studies in that anesthesia is well-known to alter neurotransmission, autonomic and cardiovascular function. Blockade of ionotropic glutamate receptors with Kyn in the region of the PVN produced a small but significant decrease in MAP and HR, suggesting that, in conscious rats, ionotropic glutamatergic transmission in the PVN participates in tonic excitatory drive to the vasculature and heart. Furthermore, the increase in MAP and HR due to bilateral blockade of either GABA_A_ receptors or inhibition of NOS indicates that in the conscious state, similar to anesthetized animals, the PVN is under tonic inhibition by both GABA via GABA_A_ receptors and by NO. The inhibitory effects of GABA and NO appear to be synergistic. Finally, these studies demonstrate for the first time that under basal conditions in the conscious rat, endogenous NO attenuates the tonic pressor effects of glutamate within the region of the PVN.

**Figure 8:**
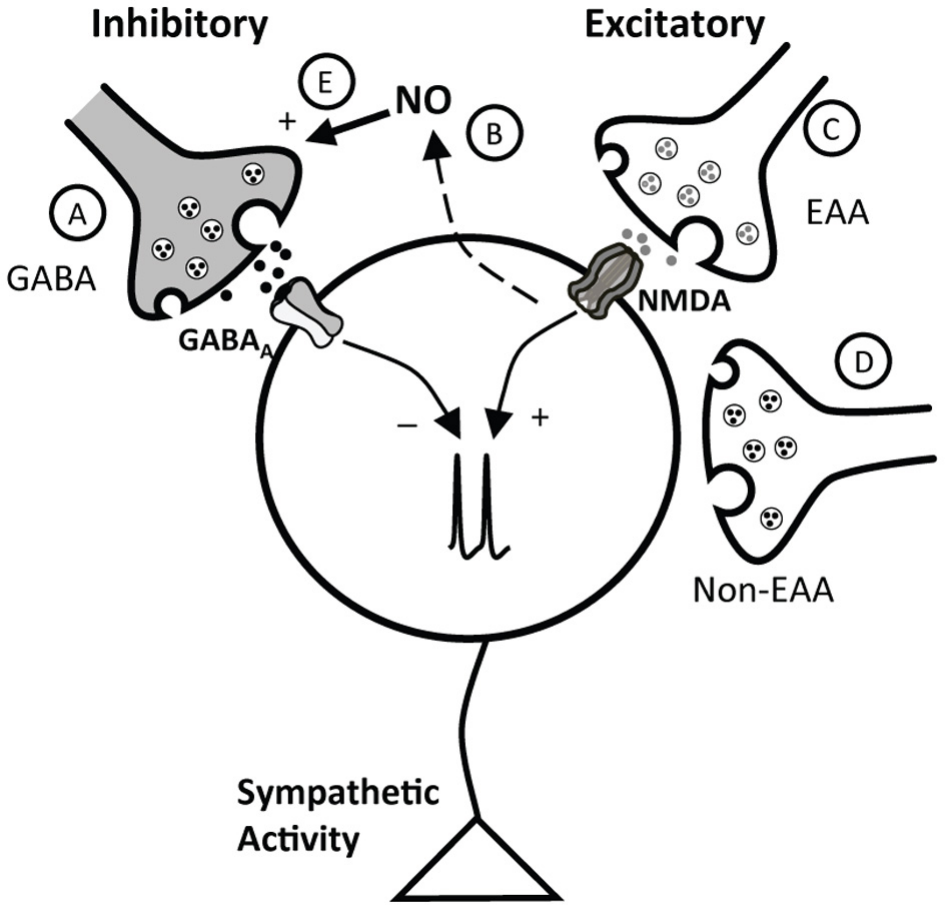
**Schematic representation of potential interactions of inhibitory and excitatory inputs to the PVN.** The cell in the schematic represents a PVN neuron involved in control of sympathetic nervous system activity. As described in the text, this neuron receives inhibitory influences due to GABA, via GABA_A_ receptors **(A)**, and NO **(B)**, which appears to exert its effects at least in part through GABA **(E)**. Excitatory inputs include both excitatory amino acid (EAA, **C**) and non-ionotropic EAA receptor mediated (non-EAA) **(D)** inputs. NO, likely acting through GABA, both blunts the response to activation of NMDA receptors, and tonically inhibits the effects of ongoing EAA excitation.

In the PVN a number of excitatory and inhibitory neurotransmitters converge to influence neuronal activity (Swanson and Sawchenko, 1983). Studies *in vitro* and in anesthetized animals suggest an interaction among glutamate, GABA, and NO in regulation of the PVN (Horn et al., 1994; Zhang and Patel, 1998; Stern, 2004). The current experiments indicate that an interaction between glutamate and GABA, as well as an interaction of NO with both glutamate and GABA, contributes to PVN control of arterial pressure in conscious rats. Taken together, the data suggest that in conscious rats the influence of the PVN on arterial pressure and HR reflects a complex balance among a variety of excitatory and inhibitory inputs. A schematic of these potential interactions is shown in Figure 8 and will be described below.

### Tonic effects of the PVN

The PVN is a critical site of neuroendocrine integration and plays an important role in modulating sympathetic tone and arterial pressure under a variety of physiological and pathophysiological conditions including heart failure, diabetes, and hypertension (Patel and Zhang, 1996; Martin and Haywood, 1998; Patel, 2000; DiCarlo et al., 2002; Felder et al., 2003; Li and Patel, 2003; Mueller et al., 2006; Heesch et al., 2009). However, its role in basal cardiovascular control is not entirely clear, although it likely reflects the integration of both excitatory and inhibitory influences. Bilateral inhibition of GABA_A_ receptors with Bic in the PVN region of conscious (Martin et al., 1991) or anesthetized (Zhang and Patel, 1998; Chen and Toney, 2003; Chen et al., 2003) rats increases MAP and HR, suggesting that the PVN is under tonic GABAergic inhibition (Figure 8A). The current study provides additional support for tonic GABAergic inhibition in conscious rats and also indicates that the region of the PVN is under tonic inhibition due to NO in the conscious state (Figure 8B). Similar to experiments in anesthetized animals (Zhang and Patel, 1998), bilateral microinjection of the NOS blocker L-NMMA increased MAP and HR. In the present study, we also investigated the contribution of EAAs in the region of the PVN to maintenance of basal arterial pressure and in the response to inhibition of GABA_A_ receptors in conscious rats. In our experiments, bilateral blockade of ionotropic glutamate receptors produced a small but significant reduction in arterial pressure and HR, suggesting that the balance of inputs is such that EAAs provide a small tonic excitatory effect within the PVN, which contributes to maintenance of basal arterial pressure and HR (Figure 8C). Similar experiments in anesthetized rats (Chen et al., 2003) and conscious rabbits (Badoer et al., 2002) showed that blockade of ionotropic EAA receptors in the PVN had no significant effect on baseline arterial pressure. The difference in these studies may be due to the presence of anesthesia, unilateral versus bilateral injections, and possible species differences. Alternatively, as previously suggested (Badoer et al., 2002), the PVN may exert both excitatory and inhibitory effects on sympathetic nerve activity, and the relative influence of these effects may vary depending on species and state of the animal. Nevertheless, the current data are consistent with the hypothesis that the relatively small tonic excitatory effects exerted by the PVN involve, at least in part, ionotropic glutamatergic transmission. Other potential sources of excitation in the PVN (Figure 8D) were not evaluated in the current experiments. However, previous studies in anesthetized rats suggest that angiotensin II also contributes to excitation of the PVN under both physiological and pathophysiological conditions (Chen and Toney, 2003; Freeman and Brooks, 2007).

### Nitric oxide and GABA

Unilateral microinjection of the NO donor SNP into the region of the PVN of conscious rats decreased MAP, and blockade of NOS with L-NMMA-induced pressor and tachycardic responses. These sympathoinhibitory effects of NO in the conscious state are consistent with previous work examining responses to SNP (Zhang and Patel, 1998) or NOS blockade (Mastelari et al., 2011) in the PVN. Other studies in conscious Wistar rats reported no effect (Hashiguchi et al., 1997) or pressor responses (Busnardo et al., 2010) to PVN microinjection of NO donors. While the reasons for this discrepancy are not clear, differences including drug concentration and strain could contribute. Taken together, the preponderance of evidence suggests that NO is an inhibitory modulator of cardiovascular control in the PVN in both conscious and anesthetized animals. Microinjection of SNP into the region of the PVN was associated with tachycardia in the present experiments rather than bradycardia as previously reported (Zhang and Patel, 1998; Wang et al., 2005). The mechanism for this tachycardia is not completely clear. It is possible that it was a baroreflex-mediated response to the decrease in arterial pressure. Alternatively, it may be a direct effect of SNP in the PVN rather than baroreflex-mediated, because although the depressor effect of SNP was diminished in the presence of Bic, the tachycardia was unchanged. Thus, the tachycardia likely was not dependent on the magnitude of the depressor response. Furthermore, it does not appear to require GABA_A_ receptors, because it was not altered by pretreatment with Bic.

Full expression of the depressor effect of NO in the PVN region of awake rats appears to require GABAergic transmission (Figure 8E), since responses to SNP were attenuated ~50% by Bic. In previous work in anesthetized animals (Zhang and Patel, 1998), Bic abolished responses to SNP. Thus, it appears that in the absence of anesthesia, a portion of the response to NO donors is independent of GABA. Another possibility may be related to the dose of Bic used. In the present study, we used a relatively low dose of Bic (1 mM). As reported by others (Martin et al., 1991), we observed that Bic injection into the PVN of conscious rats resulted in mild behavioral responses (such as licking and grooming). We found that higher doses tended to elicit locomotor activity that interfered with measurements of arterial pressure and HR. Therefore, we chose a dose within the lower range of those previously used in an effort to circumvent this concern. It is possible that this relatively low dose of Bic did not completely block GABA_A_ receptors. Although previous studies in anesthetized rats indicate that cardiovascular responses to 40 mM and 1 mM Bic microinjected into the PVN were similar (Zhang and Patel, 1998; Li et al., 2006), we cannot completely eliminate the possibility that our GABA_A_ receptor blockade was not complete in the current study. Nonetheless, it appears that in conscious rats, NO exerts primarily an inhibitory role in the PVN and this inhibition is dependent at least in part on GABA. However, it is possible that the contribution of GABA to NO-mediated effects may be less in conscious compared to anesthetized animals.

### Nitric oxide and glutamate

Excitatory responses to activation of the NMDA subtype of glutamate receptors in the region of the PVN were significantly enhanced by prior blockade of NOS. These data in conscious rats extend previous findings in anesthetized rats (Li et al., 2001) demonstrating that the excitatory effects of exogenously administered NMDA in the PVN are blunted by NO (Figure 8, dashed line). Although not specifically examined in our study, this effect of NO may also involve an effect mediated by GABA (Figure 8E) rather than modulation of glutamatergic signaling, as NO appears to increase directly glutamate release (Horn et al., 1994) and AMPA-mediated currents (Roychowdhury et al., 2006) in the PVN. The effects of NO to modulate GABA function appear to predominate, possibly due to greater tonic GABAergic input on PVN neurons.

We also examined the influence of NO on the effects of tonic endogenous ionotropic glutamate receptor mechanisms within the PVN. The NOS inhibitor L-NMMA enhanced the depressor response to bilateral inhibition of ionotropic glutamate receptors with Kyn. Importantly, the depressor response to Kyn was enhanced even though L-NMMA would be expected to increase arterial pressure over the same time period. These data suggest that in conscious rats not only does NO blunt the response to exogenously administered NMDA, but the endogenous, tonic excitatory effects of glutamate in the PVN also are under tonic negative modulation by endogenous NO. To our knowledge, this is the first demonstration of an effect of endogenous NO on tonic EAA-mediated excitation within the PVN. Since we also found that the pressor response to exogenous NMDA is potentiated following blockade of NOS, tonic effects of endogenous NO also may involve the NMDA subtype of glutamate receptors. Thus, the overall effect of NO within the PVN is inhibitory, since NO attenuates tonic excitation due to both exogenous and tonic endogenous glutamate, and facilitates inhibitory GABA neurotransmission.

### GABA and glutamate

Endogenous GABA and glutamate also appear to interact in the PVN of conscious rats. Bilateral removal of GABA_A_ receptor-mediated inhibition in the region of the PVN produced pressor and tachycardic responses, effects observed in other studies using conscious rats (Martin et al., 1991; de Abreu et al., 2009). Experiments in anesthetized animals suggest that this response is due to an unmasking of excitatory inputs, including glutamate and angiotensin II (Chen and Toney, 2003; Chen et al., 2003; Li and Pan, 2007a). In the current study, excitatory responses to GABA_A_ blockade were blunted during inhibition of ionotropic glutamate receptors. These data suggest that in the conscious rat, glutamate provides a substantial portion of the excitation that is evident when GABA_A_ inhibition is removed (Figure 8C), or glutamatergic inputs may be tonically inhibited by GABA. In this regard, following Bic in the PVN, release of glutamate and the frequency of glutamatergic EPSCs are increased (Li et al., 2006). Alternatively, GABAergic neurons in the region of the PVN may be excited tonically by glutamate. Although simultaneous disfacilitation due to Kyn could contribute to the blunted response to Bic, this most likely involves an interaction between glutamate and GABA as previously suggested (Chen et al., 2003) because the minor effects of Kyn alone are not sufficient to account for the magnitude of the diminished response. Future experiments are required to evaluate the cellular mechanisms of these interactions, as well as a potential role for other transmitters such as angiotensin II.

### Limitations

As with any study our conclusions have certain caveats that must be taken into consideration. For example, in the current study we measured arterial pressure and HR in conscious rats. It is most likely that responses to microinjections are due primarily to changes in sympathetic nervous system activity. Although this assumption is consistent with previous studies in anesthetized animals (Zhang and Patel, 1998; Li et al., 2001), numerous neural and humoral factors influence arterial pressure, and the influence of the PVN on sympathetic activity may vary depending on the specific sympathetic nerve evaluated. Therefore, future experiments are necessary to confirm the involvement of the sympathetic nervous system, including effects on sympathetic activity to specific regions, vs. other neurohumoral factors.

Also, because of non-specific damage during removal of guide cannulae, we cannot say unequivocally that all injections were centered in the PVN. However, injection sites were confined to the brain parenchyma, and the primary cardiovascular region nearest the injections was in fact the PVN. In all rats, consistent with responses in the PVN, disinhibition (Bic) or excitation (NMDA) produced pressor responses with limited behavioral activation. In addition, our coordinates are consistent with those used previously by us, in which injection sites were confirmed (Kvochina et al., 2009; King et al., 2012). Collectively, this evidence suggests that responses were due primarily to changes in neuronal activation in the region of the PVN.

## Summary

The combined data from these studies in conscious rats is consistent with previous work in anesthetized animals and suggests that the PVN is under tonic excitation due to endogenous activation of ionotropic glutamate receptors. In addition, both NO and GABA have tonic inhibitory effects in the region of the PVN in conscious rats and this inhibition masks the majority of tonic glutamatergic excitation. Importantly, we found that in conscious rats, endogenous NO appears to tonically blunt the ongoing activation of the PVN by EAAs. The nature of these tonic effects involves a complex interaction among glutamatergic, GABAergic, and NO mechanisms.

### Conflict of interest statement

The authors declare that the research was conducted in the absence of any commercial or financial relationships that could be construed as a potential conflict of interest.
